# Use of Arsenic in Constipation

**Published:** 1873-12

**Authors:** 


USE OF ARSENIC IN CONSTIPATION.—M. Isnard recommends arsenic in the treatment of constipation, especially in the case of females. Although any preparation of arsenic seems to answer; M. Isnard prefers, ordinarily, arsenious acid. Three grains being dissolved in two and one-half pints of water, a coffee-spoonful will represent one *milligramme* of the acid. A medium dose is six to ten coffee-spoonfuls daily, in three doses, preferably taken at meal-time, in water or wine, and increased gradually in quantity.—*La France Med.*

